# Energy at the Junction of the Rivers Negro and Solimões, Contributors of the Amazon River, in the Brazilian Amazon

**DOI:** 10.1155/2014/794583

**Published:** 2014-10-29

**Authors:** Alexandre Beluco, Paulo Kroeff de Souza

**Affiliations:** Instituto de Pesquisas Hidráulicas, Universidade Federal do Rio Grande do Sul, Av. Bento Gonçalves 9500, P.O. Box 15029, 91501-970 Porto Alegre, RS, Brazil

## Abstract

The Negro and Solimoes rivers join in front of the Brazilian city of Manaus to form the Amazon River. This “meeting of the waters” is a natural phenomenon of great aesthetic beauty that has been the focus of attention of researchers all over the world in various scientific fields. The waters of the Negro are darker and warmer, while the waters of the Solimoes are lighter and cooler. These waters have very different characteristics and remain without mixing, flowing side by side for several miles. Some reports indicate a temperature gradient between the waters of the order of 6°C, which can be used in conjunction with very high flow rates delivered by the two rivers, with a heat engine operating on a thermodynamic cycle to provide electricity. This review paper identifies this energy resource and presents a preliminary assessment of the potential for power generation. A realistic assessment of the potential points to an available power of about 1 GW. It is clear that further studies are needed to accurately assess the available thermal gradient and its variation over time, to move forward in the design of the power converter, and to establish an appropriate location for a power plant.

## 1. Introduction

The Amazon basin has a drainage area of more than six million square kilometers, producing an average flow of around two hundred thousand cubic meters per second, ranging from about one hundred thousand cubic meters per second in the dry season and about three hundred thousand cubic meters per second in times of flood. The average flow rate corresponds to about one-fifth of all the fresh water that reaches the oceans coming from the continents. The river, stretching over more than six thousand kilometers from the foot of the Andes to its mouth in the Atlantic Ocean, can be several hundred meters wide and can even reach a hundred feet deep. The city of Manaus (http://goo.gl/maps/YnYji) is located around 1500 km from the mouth and is only sixty feet above sea level, leading to a slope that varies from thence to the mouth, between one and two inches per mile, between droughts and floods.

The Negro and Solimões rivers meet in front of the city of Manaus (approximately 3°S, 60°W (http://goo.gl/maps/KBLYF)), which is the capital of the Brazilian state of Amazonas (http://goo.gl/maps/64k1o), to form the Amazon River. The phenomenon of the separation of the waters of the two rivers is well known and occurs for several kilometers along the Amazon River bed and such separation is attributed to several factors. For a long time, it was thought that the two rivers ran side by side, but the French Brazilian research program “Hydrology and Geochemistry of the Amazon Basin” [1] showed that the waters of the river Solimões, which is most copious, move beside and below the waters of the river Negro, until fully mixed.

Laraque et al. [2] mention that a partial mixing of the waters of the two rivers is already evident from about twelve kilometers further from the initial meeting, but complete mixing occurs only about a hundred kilometers beyond the meeting point of two rivers. A quick visit to the NASA website or Google Maps allows a preview of the meeting of the waters (http://www.nasa.gov/multimedia/imagegallery/image_feature_577.html) and navigation on Google Maps allows the observation that the interface between the waters of the two rivers persists until Itacoatiara (http://goo.gl/maps/el1CP), about 150 kilometers down along the Amazon River.

**Figure 1:**
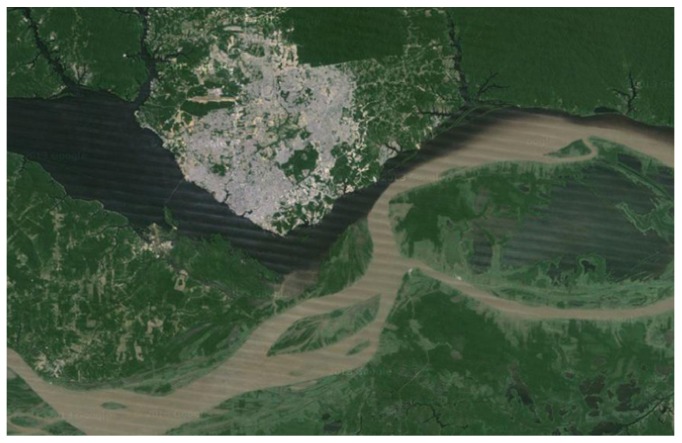
The region of the meeting of the waters. (Source: Google Maps at http://goo.gl/maps/JXvZx.)

Figure 1 shows the region of the “meeting of the waters,” as it is known. The satellite image shows the city of Manaus and the junction of the dark waters of the Negro River, coming from the northwest, and the muddy waters of the Solimões River, coming from the southwest, to form the Amazon River, flowing eastward.

Laraque [1] presents a very interesting comment, which reports that local fishermen utilize the meeting of the waters and the clear interface between the black waters of the Negro River and the muddy waters of the Solimões River to create a trap by placing the fish between the interface of the waters of the two rivers and their fishing nets.

The project mentioned above revealed that the waters of the Negro and Solimões rivers have different speeds (respectively, 0.3 m/s and 1.0 m/s), different conductivities (respectively, 8 *μ*S/cm and 80 *μ*S/cm at 25°C), different turbidity values (respectively, 5 NTU and 80 NTU), different pH values (respectively, 5.5 and 7.0), and different temperatures, indicating a difference of 1°C.

Tao et al. [3] and Aucour et al. [4] provide an extensive study on the mixing of the waters of the two rivers in the region of their meeting, presenting measurements of concentration of various elements in various sections throughout the meeting of the waters, without conducting, however, temperature measurements. The authors report that complete mixing is achieved only about 25 km downstream from the meeting point and massive amounts of Ca, Mg, Si, Fe, and Al present in particulate materials are lost without lateral contributions of these elements by groundwater or floodplain ponds. In the dissolved fraction, Al behaves conservatively, while Mn has an increased concentration downstream. The work also indicates a large loss of dissolved organic carbon in the mixture of waters of the two rivers.

Aucour et al. [4] present vertical profiles in cross sections along the test region of the meeting of the waters, which together with the data provided by Silva and Pecly [5] and data obtained from the database Hydroweb allow knowing quite well the shape of the cross sections of the rivers Negro and Solimões and Amazonas and the vertical distribution of speeds in the region of the meeting of the waters.

The waters of the Negro River, as indicated by its name, are darker and warmer, usually treated in the literature as “black water,” while the waters of the Solimões River are more muddy and cooler, treated as “clear water” or “white water.” This difference appears even as a difference in the movement of air masses [6, 7].

Several national websites (http://www.descobrindooamazonas.webs.com/encontrodasguas.htm) (http://www.portalamazonia.com.br/secao/amazoniadeaz/interna.php?id=242) related to tourism and natural heritage, approaching the “meeting of the waters,” mention temperatures of 22°C to 24°C for Negro and 28°C to 30°C for Solimões, a temperature difference of 4°C to 8°C. Also, they mention speed of 2 km/h for the waters of the Negro River and 4 to 6 km/h for the waters of the Solimões River.

The temperature difference between the waters of the two rivers can be harnessed to produce electricity through a system similar to that used in geothermal plants or plants that harness the thermal gradient of the ocean waters. A technological breakthrough to generate energy from small temperature gradients will obviously be necessary.

A project of the 70s [8] prepared by the National Institute for Amazonian Research (INPA) and the State University of Campinas (UNICAMP) to obtain funds from the Financier of Studies and Projects (FINEP) to quantify the difference in temperature between the rivers Negro and Solimões foresaw the development of temperature meters in depth and pointed out a temperature difference of 7°C. The project was not funded by FINEP apparently because it was not associated with the development of a strategy for the transport of energy generated outside the region, for the major consuming centers of the country.

This project presented an initial estimate of maximum available power of about 15 GW and a specific cost estimate of US$ 0.28/MW installed. The project compares this estimate with the cost of ocean thermal plants, from US$ 0.63/MW installed, and assigns the difference of these two values to greater difficulties associated with this type of use. The OTEC plants are subject to storms and corrosion and are installed on floating vessels and demand a reasonable amount of energy for pumping water from great depths. The author further argues that the availability of this power plant would allow the use of large-scale deposits of aluminum in the Amazon region as well as the production of ammonia, nitrogen fertilizers, and hydrogen.

Brazil has faced problems over the past three decades in the energy sector, first with the oil crisis and then with the lack of installed power and energy stored in reservoirs to meet economic growth evidenced in the 90s. The difficulties have always encouraged projects of renewable resources and currently have increased the participation of wind farms and even of photovoltaic systems in the energy matrix. However, this energy resource in the Amazon was not considered even in texts of nongovernmental organizations [9].

This paper aims to present this energy resource for discussion and present a preliminary evaluation of the potential available for power generation from this untapped resource. It is clear that the temperature gradient between the rivers Negro and Solimões is significantly lower than the gradients available in geothermal and ocean thermal plants, but it is possible to provide reasonable power since the flows available for use are fairly high. This paper is a very preliminary study, based on theoretical considerations and without any concerns related to technological issues, intending only to present this previously unknown energetic potential.

The newness of the subject justified the inclusion of the term “river thermal energy” in the keywords of this paper.

## 2. Energy in a Small Thermal Gradient

The thermal gradient between the waters of the Negro and Solimões rivers can be harnessed to generate energy with a thermodynamic cycle similar to that used in thermal power plants. The difference appears in the intensity of this thermal gradient, which is very small, of the order of 5°C to 8°C.

Plants that use naturally available thermal gradients, as in geothermal and ocean thermal power plants, generate energy from thermal gradients with a few tens of degrees Celsius. Several authors [10, 11] say that it is not economically feasible to generate power thermal gradients less than 15°C.

In fact, power generation from thermal gradients is so limited and the use of high flow rates will require research work for the selection of a suitable working fluid and the development of heat exchangers and the design of a plant that is appropriate to prevailing environmental concerns.

**Figure 2:**
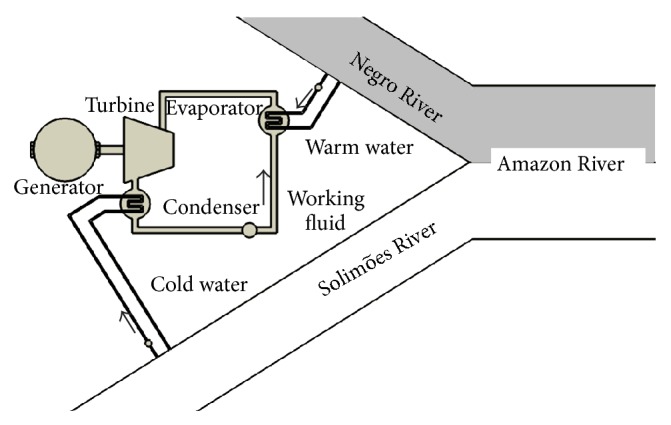
Schematic representation of the power converter.

Figure 2 shows schematically the river thermal power plant placed near the meeting of the waters. The warm source is maintained by the waters of the Negro and the cold source is maintained with the waters of the Solimões River. The figure shows the water being returned to its rivers, after passing through the heat exchanger, but this issue is discussed later.

The mechanical power *P*_mec_ (W) that can be extracted in the system is given by (1), where *ρ* (kg/m^3^) is the specific mass of water, *c* (J/kg·K) is the specific heat capacity at constant pressure, *Q* (m^3^/s) is the flow rate, *T*_*w*_ (K) is the temperature of the warm source, *T*_*c*_ (K) is the temperature of the cold source, and *η*[1] is the efficiency. Consider

(1) $$\begin{array}{c} P_{\text{mec}}=\rho cQ\left(T_{w}-T_{c}\right)\eta . \end{array}$$

This power is ideal and actual machines operate closer to a Rankine cycle. The performance in a Carnot cycle is given by (2), but this limit would be obtained only in a conservative system. A more realistic measure of efficiency can be obtained with (3), which was obtained considering that the heat transfer processes are irreversible. One has

(2) $$\begin{array}{c} \eta_{\text{carnot}}=\frac{T_{w}-T_{c}}{T_{w}}, \end{array}$$

(3) $$\begin{array}{c} \eta =1-\sqrt{\frac{T_{c}}{T_{w}}}. \end{array}$$

The exploitation of a gradient of 6°C between 32°C and 26°C, for example, would result in an efficiency of 0.998% and 8°C gradient between 33°C and 25°C would result in an efficiency of 1.333%. Very low efficiency values could be realized only under the conditions found in the Amazon, with large flows.

Assuming smaller temperature gradients throughout most of the year, 3°C between 30°C and 27°C would result in an efficiency of 0.499% and 3°C gradient between 23°C and 20°C would result in an efficiency of 0.511%. A temperature gradient of 2°C between 30°C and 28°C would result in 0.332% and between 22°C and 20°C would result in 0.341%.

The hypothetical situation of the use of 1,000 m^3^/s, with a temperature gradient of 6°C, would lead to a theoretical maximum of about 240 MW of mechanical power available for conversion to electricity. As shown in the next chapter, the Negro river flow varies between 30,000 m^3^/s and 10,000 m^3^/s, much higher than the value considered.

The pumping of 1.000 m^3^/s of water over 1,000 m with two pipes with diameters of 1 m would result in a power consumption of about 10 kW. The total energy consumption for pumping, considering warm water, cold water, and working fluid, would not exceed 2% of the power of 500 MW, available with this flow.

The design problem of the thermodynamic cycle [12–14], operating with a thermal gradient so small [15–18], the working fluid selection [19–21], design of heat exchangers, and the location of the system components are very similar to those found in geothermal [22, 23] and ocean thermal energy conversion systems [24, 25].

## 3. Characterization of the Meeting of the Rivers Negro and Solimões

The evaluation of the available potential requires knowledge of river flows and water temperatures of rivers and their variations over a year. This communication presents a preliminary assessment, based on data obtained from the literature.

**Figure 3:**
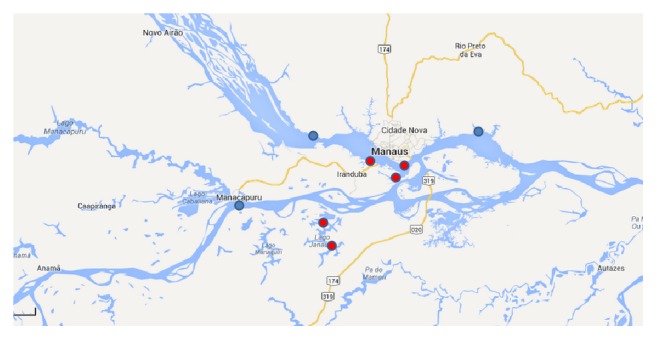
Locations of the data sources. In blue, stream gage stations. In red, temperature measurements.

The hydrological behavior in the region of the “meeting of the waters” can be characterized with data from three fluviometric stations, available online. One of the stations, Jatuarana (station Jatuarana, code 15030000, Amazonas River, Manaus, State of Amazonas, responsibility of National Agency of Waters [26], with drainage area of 2.854.286 km^2^), is located in the Amazon River, downstream from the city of Manaus and the junction of the rivers. The other two stations are located one on the Negro River, Serrinha (station Serrinha, code 14420000, Negro River, Santa Isabel do Rio Negro, State of Amazonas, responsibility of National Agency of Waters [26], with drainage area of 279.945 km^2^), and the other on the Solimões, Manacapuru (station Manacapuru, code 14100000, Solimões River, Manaus, State of Amazonas, responsibility of National Agency of Waters [26], with drainage area of 2.147.736 km^2^).  Figure 3 shows the location of these stations.

According to Cappelaere et al. [27] and Guyot et al. [28], the water level in the city of Manaus reaches its maximum annual value between May and July and lasts for several weeks around this maximum value. Manaus is situated immediately on the backwater of the “meeting of the waters” and waterline depends much more on the flow of the Amazon River (the sum of the flows of the two rivers) than on the flow of the Negro River.

The waterline can also be influenced by the flow rate of the Madeira River, whose mouth in the Amazon River is less than 200 km downstream.

**Figure 4:**
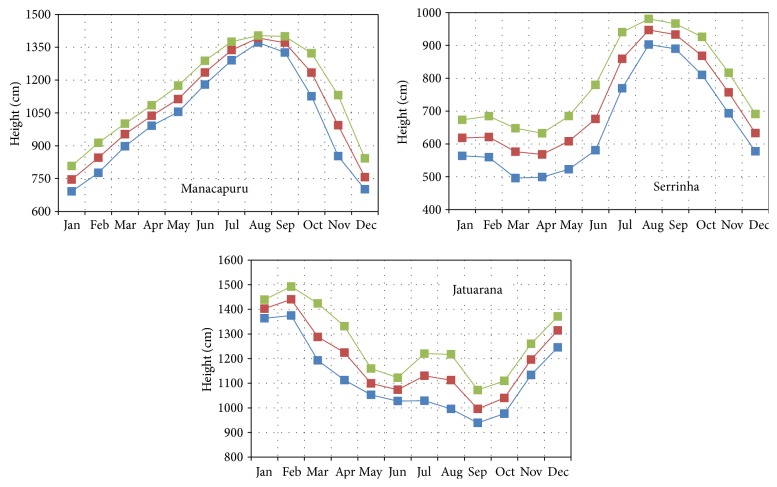
Maximum, average, and minimum levels.

Looking at the data of these three stations, floods occur between May and August and the dry season extends from approximately the months of October and February.  Figure 4 shows the minimum, average, and maximum historical levels in Manacapuru, Serrinha, and Jatuarana, corresponding, respectively, to series from January 1973 to December 2011, January 1978 to December 2005, and January 1978 to December 2005.

In Solimões, there was variation between the maximum and minimum values of the average quota of 0,865 m; at the station in Negro River, there was a variation of 0.365 m, while at the Amazon River there was a variation of 0.829 m.

**Figure 5:**
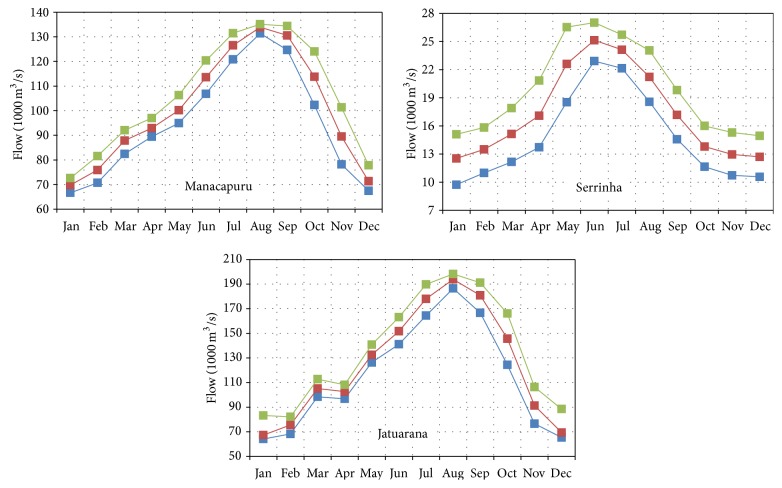
Maximum, average, and minimum discharges.

Figure 5 shows the discharge data, for series from January 1974 to December 1985, January 1978 to December 2012, and January 2006 to December 2011. In Solimões, the maximum discharge was 137.462 m^3^/s, in June, and the minimum was 68.218 m^3^/s in November. In Negro, the maximum discharge was 26.108 m^3^/s in June, and the minimum was 10.883 m^3^/s in February. In the Amazon River, the highest discharge was 165.159 m^3^/s in June, and the minimum was 83.549 m^3^/s in November.

It is also observed that the maximum values of monthly mean discharges were 160.444 m^3^/s, 32.028 m^3^/s, and 193.124 m^3^/s, respectively, for stations in the Solimões River, the Negro River, and the Amazon River, and the minimum values were 54.124 m^3^/s, 3.599 m^3^/s, and 56.303 m^3^/s, respectively, for the three stations.

**Figure 6:**
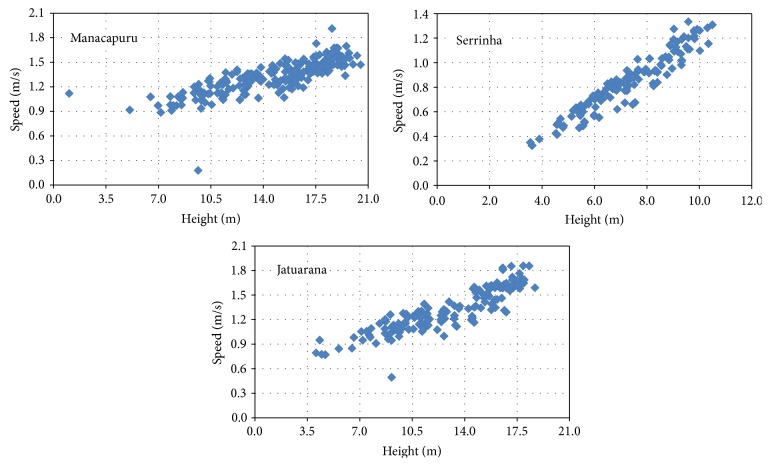
Average speeds.

Figure 6 shows the cross-sectional profiles. The cross section of the Solimões River in Manacapuru has an average width of 3,300 m and depths in the central portion that can reach 40 m. In the Negro River in Serrinha, the average width is 1900 m and depths can reach about 20 m. The cross section of the Amazon River in Jatuarana has an average width of 2800 m and depths in the central portion that can reach 60 m.

**Figure 7:**
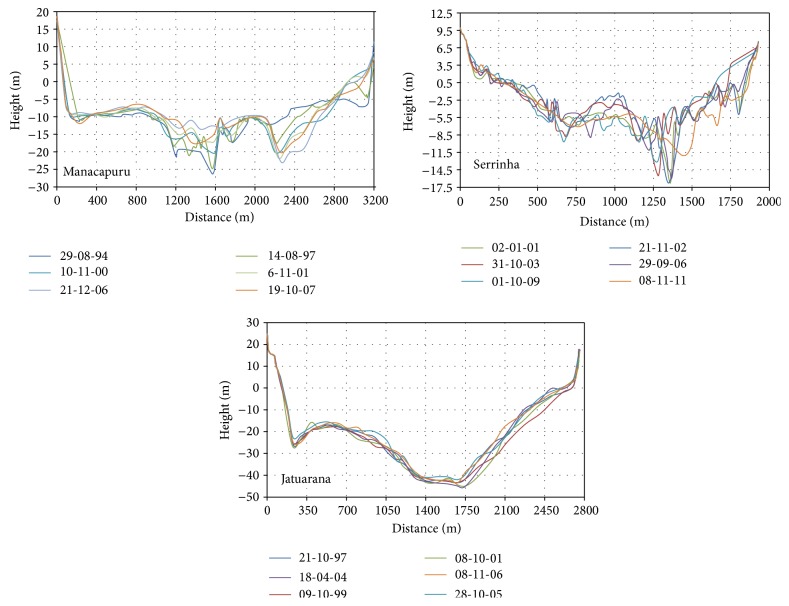
Cross-sectional profiles for different dates.

Figure 7 shows average speeds estimates for the three stations. The estimated average speeds along the flow section varied between 0.90 m/s and 1.90 m/s, between 0.32 m/s and 1.30 m/s, and between 0.50 m/s and 1.85 m/s at stations, respectively, of the Solimões River, the Negro River, and the Amazon River.

The hydrological characteristics of the region of the meeting of the waters must be evaluated in order to determine the real energetic potential available for this type of energy resource, in order to know the available energy resource and to determine the best location for the power plant.

**Figure 8:**
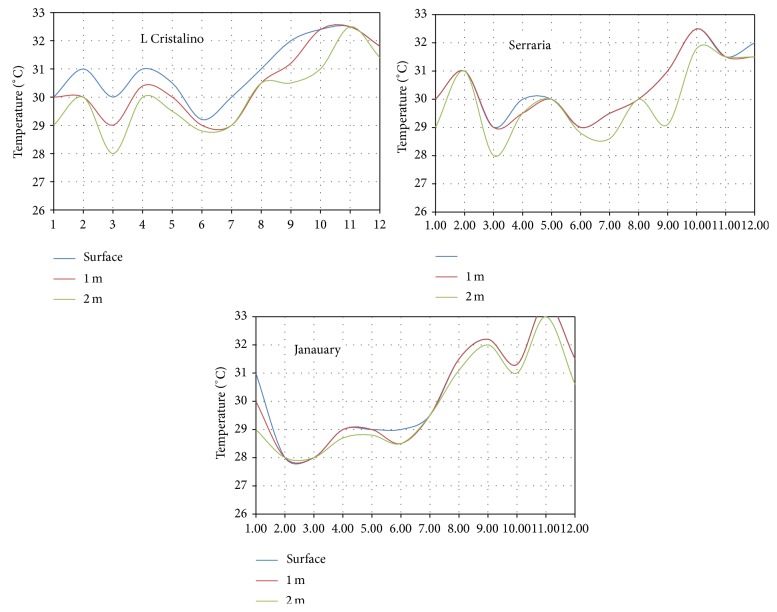
Monthly average temperatures in the Negro River.

**Figure 9:**
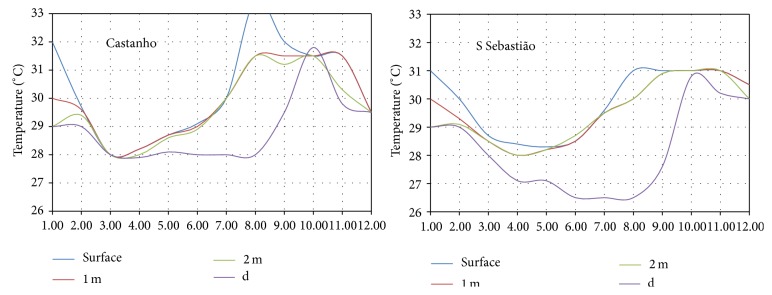
Monthly average temperatures in the Solimões River.

The behavior of the temperature in the region of the meeting of the waters can be determined from the temperature data at five sites in the region, obtained from the work of Fonseca [29].  Figure 3 shows the location of the five sites.  Figure 8 shows monthly average values of temperature for three stations at Negro River while Figure 9 shows values for two stations at Solimões River.

**Figure 10:**
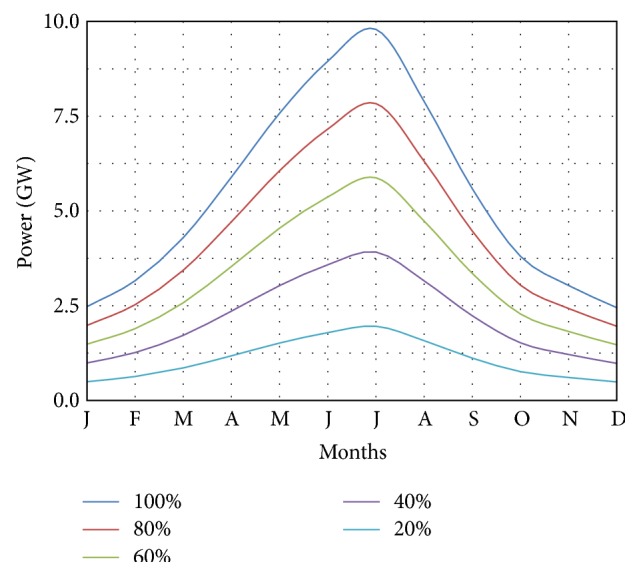
Power that can be provided depending on the utilized portion of the flow of the Negro River.

In Figure 9, there are monthly average temperatures for the surface, to 1 meter and 2 meters deep. In Figure 10, there are monthly average temperatures for the surface to 1 meter to 2 meters deep and there are still temperature values obtained at the bottom of the Solimões River.

The stations on the Negro River are Lago Cristalino, Serraria, and Janauary. The stations on the Solimões River are Castanho and São Sebastião. None of these measurement points are located near to places of interest to the water intake for the generating system and these points are located away from the main flow of the two rivers, in sites with low speed water displacement.

These measurements were not made with the intention of evaluating the energetic potential and were inserted into a project that had other goals. However, these temperature values allow a better understanding of the behaviour of monthly average temperatures over a year.

In fact, the temperatures in these graphs do not differ substantially from each other. However, it can be seen that the temperatures obtained from the Solimões River are lower and that the temperatures obtained in the bottom present reasonable thermal gradients in relation to the observed temperatures on the surface of Negro river.

The maintenance of the cold source should be done with taking water deep into the Solimões River, approximately as it is done with the cold source in ocean thermal power plants. There are reports of people of the region about chunks of ice floating in the waters of the Solimões River in some periods of the year, coming obviously of the highest points of the basin, located in the Andes.

The behavior of the thermal gradient between the waters of the two rivers over a year needs to be accurately determined on a project focused on power generation.

A comprehensive study of the flow and thermal gradient available is needed to accurately provide the available energetic potential and provide information for the leasing of a river power plant and establish appropriate locations for water intake to keep the heat exchangers.

## 4. Energetic Potential

The Negro River has smaller flow rate than the Solimões River and can be considered as a reference in the following remarks. Clearly, equivalent amounts of water of the Solimões River are also necessary.

Considering a hypothetical situation in which 100% of the flow of the Negro River was harnessed for power generation, an average of 19,919.2 m^3^/s, a total of 47,306.03 GWh per year, with a maximum of almost 20 GW and an average of 9,79 GW could be made available.

Figure 10 shows the mechanical power that can be provided depending on the utilized portion of the Negro. These results already take into account the power consumption for pumping.

Considering the use of 20% of the flow of the Negro River for power generation, equivalent to an average of 3,984 m^3^/s, a total of 9,461 GWh per year, with a maximum of 1.96 GW and an average of 1.08 GW, could be made available.

Similarly, considering the use of 10% of the flow of the Negro River for power generation, equivalent to an average of 1,992 m^3^/s, a total of 541 GWh per year, with a maximum of 0.98 GW and an average of 0.54 GW, could be made available.

Note that these values would be available for conversion into mechanical energy and then into electricity. The performances of converting equipment were not considered in these projections because they are considered outside the scope of this work.

The use of larger portions of the flow rate of the Negro River will certainly involve larger hydraulic structures and potentially devastating environmental impacts.

But the use of portions of the flow rate of the Negro of approximately 10% to 20% seems much more reasonable while still providing significant amounts of power.

The power currently installed in Brazil is 124.33 GW [30] and the addition of 0.54 GW to 1.08 GW, as discussed above, in a region that is not connected to the Brazilian energy system would be a key support for the development of the region and certainly the ultimate justification for their interconnection.

## 5. Concerns about Environmental Issues

The “meeting of the waters” was listed (http://portal.iphan.gov.br/portal/montarDetalheConteudo.do?id=16243&sigla=Noticia&retorno=detalheNoticia) by the Brazilian Institute of National Historical and Artistic Heritage (http://portal.iphan.gov.br/) (IPHAN) in 2010 and there is a request to be listed as heritage of humanity by UNESCO. This tipping has serious implications on the possibility of power generation from thermal gradient between the two rivers.

The size of the available energetic potential and the need for energy supplies can transform this energy resource into a strategic asset. That would be the only way to reverse this tipping, at least partially, for its use for power generation.

In the case of the use of this energy resource to become a reality, many environmental concerns should be taken into account. The most obvious is that the amount of energy generated will establish the proportion that the phenomenon of the meeting of the waters will be dissipated.

The use of 10% to 20% of the flow rate of the Negro, as discussed above, also will play a far-reaching impact on the natural phenomenon. As a result, it may be important to properly choose a location for return of waters used in heat exchangers. This water may even be returned few miles downstream to reduce the impact on tourism.

## 6. Final Remarks

This review paper presented an energy resource that has not been discussed or considered as an alternative, present in the temperature gradient of the waters of the Negro and Solimões rivers, where they join to form the Amazon River.

The thermal gradient is very small, of approximately 5°C to 8°C, allowing the generation of energy just because of the high flow rates available in the Amazon region.

It is estimated that the use of 20% of the waters of the Negro River, and equivalent amount of water of the Solimões River, provides about annual average of 1.08 GW of gross energy.

This amount of energy available in the Amazon has fundamental role in the development of the region and would be definitive for interconnection to the Brazilian energy system.

Obviously, several environmental concerns should be taken into account. Concerns that are beyond the scope of this paper only intended to present this energy resource. Environmental concerns such as the relationship between the energy and the extracted portion of the phenomenon of the meeting of the waters should be preserved and any other related to undertaking of power generation. Recently, the meeting of the waters was listed as national natural heritage and this prevents the generation of energy from this resource unless its use assumes a strategic character that transcends heritage preservation.
